# Metagenomic Insights into the Diverse Antibiotic Resistome of Non-Migratory Corvidae Species on the Qinghai–Tibetan Plateau

**DOI:** 10.3390/vetsci12040297

**Published:** 2025-03-23

**Authors:** You Wang, Quanchao Cui, Yuliang Hou, Shunfu He, Wenxin Zhao, Zhuoma Lancuo, Kirill Sharshov, Wen Wang

**Affiliations:** 1 State Key Laboratory of Plateau Ecology and Agriculture, Qinghai University, Xining 810016, China; wy09122289@163.com (Y.W.); cqc5614@163.com (Q.C.); houyuliang163163@163.com (Y.H.); lczm1980@163.com (Z.L.); 2 College of Eco-Environmental Engineering, Qinghai University, Xining 810016, China; 3 Xining Wildlife Park of Qinghai Province, Xining 810016, China; heshunfu_xnzoo@126.com (S.H.); 15297013980@163.com (W.Z.); 4 Federal Research Center of Fundamental and Translational Medicine, Novosibirsk 630117, Russia; sharshov@yandex.ru

**Keywords:** corvidae, avian microbiome, gut microbiota, metagenomic sequencing, antibiotic resistance genes

## Abstract

Antibiotic resistance represents a global health crisis with far-reaching implications, impacting multiple domains concurrently, including human health, animal health, and the natural environment. Wild birds were identified as carriers and disseminators of antibiotic-resistant bacteria (ARB) and their associated antibiotic resistance genes (ARGs). A majority of studies in this area have concentrated on migratory birds as carriers for the spread of antibiotic resistance over long distances. However, there has been scant research on the resistome of non-migratory Corvidae species that heavily overlap with human activities, which limits our understanding of antibiotic resistance in these birds and hinders the development of effective management strategies. This study employed a metagenomics approach to examine the characteristics of ARGs and mobile genetic elements (MGEs) in five common Corvidae species inhabiting the Qinghai–Tibetan Plateau. The ARGs were classified into 20 major types and 567 subtypes. Notably, ARGs associated with multidrug resistance, including to macrolide–lincosamide–streptogramins, tetracyclines, beta-lactam, and bacitracin, were particularly abundant, with the subtypes *acrB*, *bacA*, *macB*, *class C beta-lactamase*, and *tetA* being especially prevalent. A total of 5 types of MGEs (166 subtypes) were identified across five groups of crows, and transposase genes, which indicated the presence of transposons, were identified as the most abundant type of MGEs. Moreover, some common opportunistic pathogens were identified as potential hosts for these ARGs and MGEs. Procrustes analysis and co-occurrence network analysis showed that the composition of the gut microbiota shaped the ARGs and MGEs, indicating a substantial association between these factors. The primary resistance mechanisms of ARGs in crows were identified as multidrug efflux pumps, alteration of antibiotic targets, and enzymatic inactivation. High-risk ARGs which were found to potentially pose significant risks to public health were also analyzed and resulted in the identification of 81 Rank I and 47 Rank II ARGs. Overall, our study offers a comprehensive characterization of the resistome in wild Corvidae species, enhancing our understanding of the potential public health risks associated with these birds.

## Simple Summary

This study explores antibiotic resistance in five common Corvidae species from the Qinghai–Tibetan Plateau using metagenomics. It identifies 20 types and 567 subtypes of antibiotic resistance genes (ARGs), with notable abundances in multidrug, macrolide–lincosamide–streptogramin, tetracycline, beta-lactam, and bacitracin resistance genes, particularly *acrB*, *bacA*, *macB*, *class C beta-lactamase*, and *tetA*. Among the 5 categories of mobile genetic elements (MGEs) that were identified (comprising 166 subtypes), transposase genes, which indicate the presence of transposons, were the most prevalent. Opportunistic pathogens were identified as potential hosts for ARGs and MGEs. Gut microbiota composition significantly influenced ARG and MGE profiles, based on Procrustes and co-occurrence network analysis. Primary resistance mechanisms included multidrug efflux pumps, alteration of antibiotic targets, and enzymatic inactivation. This study identified 81 Rank I and 47 Rank II high-risk ARGs posing public health risks. This work enhances our understanding of the resistome in wild Corvidae and the associated public health implications.

## 1. Introduction

One of the most significant public health challenges in the 21st century is the emergence of antibiotic resistance. A UK government-commissioned review on antimicrobial resistance predicts that, by 2050, the annual number of deaths attributable to antimicrobial resistance could reach 10 million globally [1]. China ranks among the top countries in the world for antibiotic production and consumption and is also the largest producer of animal products globally. Each year, tens of thousands of tons of antibiotics are produced in China, with approximately half being used in livestock and poultry production [2]. The misuse of antibiotics in livestock farming has gradually heightened bacterial resistance levels, expanded the range of resistance, and resulted in the development of highly resistant bacteria and multidrug-resistant strains [3]. In severe cases, this has led to the emergence of “superbugs”, which pose significant challenges to the sustainable development of the livestock industry [4]. Research has demonstrated that antibiotics are frequently detected in various environmental media in China, with detection rates in soil, surface water, and coastal waters reaching 100%, 98.0%, and 96.4%, respectively [5]. On the one hand, the inappropriate or excessive use of antimicrobial agents imposes selective pressure on pathogenic bacteria; on the other hand, bacterial cross-resistance and clonal spread make it increasingly challenging to prevent and treat multidrug-resistant bacteria in clinical settings. This situation has resulted in severe societal harm and a growing medical burden. Research has shown that antimicrobial use in food-producing animals worldwide reached about 131,109 tons in 2013 and is expected to rise to 200,235 tons by 2030 [6].

Wild animals serve as important sources and reservoirs of ARGs, with invertebrates [7], birds [8], and mammals [9,10] all contributing to their dissemination. Among these, birds are particularly notable due to their ability to migrate long distances, as well as their unique diets, lifestyles, and complex physiological characteristics [11,12]. Each year, billions of birds undertake long-distance migrations between their wintering and breeding grounds, with migration routes spanning every continent. This extensive migratory pattern facilitates the worldwide spread of ARGs and ARB [13]. Studies have shown that wild birds, such as waterbirds [14,15], *Passer domesticus* [16], and Corvidae species [17], carry various ARGs and ARB. Wild birds, which often inhabit polluted environments, are considered reservoirs and potential dispersers of ARGs. For instance, Oravcová et al. isolated 75 vancomycin-resistant *Enterococcus faecium* (VREfm) strains from wild Corvidae species in Europe and Canada [17]. The findings suggested that the types of *vanA* plasmids carried by VREfm in wild Corvidae birds were influenced by human activities, making these birds potential vectors for the global spread of vancomycin resistance. Although wild birds did not directly interact with antibiotics, evidence indicated that they could acquire and spread resistant bacteria through environmental exposure and thrive in human-altered habitats. Research indicated that wild birds in human-altered environments harbored a greater number of ARGs compared to those in remote regions [18].

As one of the most biodiverse areas on Earth, the Qinghai–Tibetan Plateau played a vital role as an ecological security shield for China and Asia and attracted significant attention in global biodiversity research. Recognized for its role as a natural habitat for rare wildlife and a genetic reservoir for plateau species, this region has long been a central focus of numerous scientific investigations [19]. A study found that yaks in the Qinghai–Tibetan plateau carry *Clostridium perfringens* isolates harboring broad-spectrum ARGs, indicating a significant risk of antimicrobial resistance transmission and posing challenges to public health [20]. As omnivores, Corvidae birds exhibit a remarkable adaptability in their feeding habits, consuming a diverse diet that includes grains, fruits, insects, carrion, and even food scavenged from urban garbage dumps [21]. This dietary flexibility has been identified as a key factor contributing to their survival. Furthermore, previous studies demonstrated that crows employ observational learning to locate and access food resources, showcasing their advanced social and cognitive skills, which facilitate their ability to thrive in urban environments [22]. Given their frequent interactions with human-altered environments, crows may also serve as important carriers or disseminators of ARGs. While studying the gut resistome of wild birds, especially species that inhabit areas overlapping with human activity, holds great ecological importance, this area of research has received limited attention. Our study seeks to fill this gap by examining the prevalence and diversity of ARGs in crow populations, offering new understanding into how these birds contribute to the spread of antibiotic resistance.

## 2. Materials and Methods

### 2.1. Ethics Statement

This study adhered to the guidelines for the care and use of experimental animals set by the Ministry of Science and Technology of the People’s Republic of China (Approval No.: 2006-398). The research protocol was evaluated and approved by the Ethics Committee of Qinghai University.

### 2.2. Samples Collection

**Figure 1:**
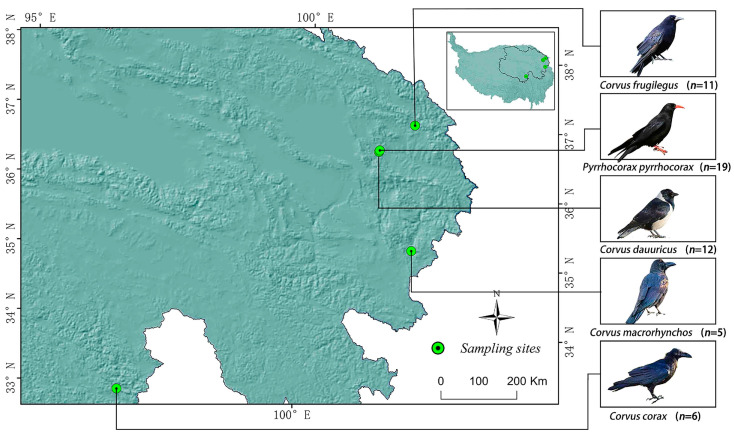
Bird sampling site map.

In this study, 42 individuals from three crow species were captured in a specific region of Qinghai Province, China (Figure 1). The species involved were red-billed choughs (*Pyrrhocorax pyrrhocorax*), Daurian jackdaws (*Corvus dauuricus*), and rooks (*Corvus frugilegus*). The samples were divided into three groups: RBC group (19 individuals), DJ group (12 individuals), and RK group (11 individuals). Following transport to the laboratory, each individual underwent dissection, during which the intestines were carefully excised and their contents expressed. All intestinal samples were preserved at −80 °C for subsequent analysis. In addition, fresh fecal material was collected from two additional crow species—large-billed crows (*Corvus macrorhynchos*) and northern ravens (*Corvus corax*)—with sample sizes of *n* = 5 and *n* = 6, respectively, and designated as the LBC and NR groups (Figure 1). The fecal samples were collected promptly after defecation by the birds, under the supervision of field personnel, and were quickly frozen with liquid nitrogen to preserve the biological material’s integrity. Subsequently, all samples were transported to the laboratory and stored at −80 °C for subsequent analysis.

### 2.3. DNA Extraction and Metagenomic Sequencing

The extraction of genomic DNA from all 53 samples was performed using the Qiagen QIAamp DNA Stool Mini Kit (Qiagen, Hilden, Germany), following the protocol provided by the manufacturer. To ensure the removal of any RNA contamination, the DNA extracts underwent treatment with RNase that was free of DNase activity. DNA concentrations were quantified using a Qubit 2.0 Fluorometer (Invitrogen, Waltham, MA, USA). The purity of the extracted DNA was assessed by measuring the absorbance ratios at 260/280 nm and 260/230 nm with a Nanodrop spectrophotometer (Thermo Scientific, Waltham, MA, USA). For metagenomic analysis, shotgun sequencing libraries were constructed and sequenced by Shanghai Biozeron Biological Technology Co., Ltd. (Shanghai, China) The procedure included building paired-end (PE) libraries with an insert size of 450 base pairs for each sample. Subsequently, high-throughput sequencing was performed on the Illumina NovaSeq 6000 platform, generating PE reads that were 150 base pairs long on each end.

### 2.4. Raw Data Processing and Taxonomy Profiling

For all bioinformatics software and tools utilized in this research, default parameters were applied unless specific adjustments are stated. High-quality reads were achieved by eliminating low-quality sequences that contained ambiguous “N” bases, adapter remnants, and potential contamination from the host DNA. The reference genome used for identifying host contamination was that of the large-billed crow (*Corvus macrorhynchos*), specifically available from the NCBI Trace Archive (https://trace.ncbi.nlm.nih.gov/Traces/?view=run_browser&acc=DRR250114&displad=metadata, accessed on 1 October 2024) (accession number DRR250114). The filtering process employed Trimmomatic version 0.39 [23] and Bowtie2 version 2.4.1 [24], integrated within the KneadData workflow (https://github.com/biobakery/kneaddata, accessed on 1 October 2024) to ensure thorough preprocessing of the sequencing data. Single-sample assemblies were carried out using MEGAHIT (version 1.2.9) [25]. Subsequently, gene prediction was carried out on the assembled contigs that exceeded 500 base pairs in length, utilizing Prodigal (version 2.6) [26]. Taxonomic information at all levels-from phylum down to species-was obtained by aligning the unigenes against the NCBI NR database using Diamond (version 0.9.22) [27]. The alignment parameters were set to an e-value threshold of ≤1 × 10^−5^ and a score threshold of ≥60.

### 2.5. ARG and MGE Annotation and Quantification

Based on the high-quality metagenomic reads, the identification and classification of ARGs into various types and subtypes were conducted using the ARGs-OAP pipeline version 2.0, with the SARG database (Structured ARG Reference Database) [28] serving as the reference. ARGs were annotated using the following criteria: an e-value threshold of 1 × 10^−7^, a minimum amino acid similarity of 80%, and a hit length coverage of 75%. Comparisons of ARGs among groups were conducted at both the type and subtype levels, with results expressed in unit “ppm”, defined as one read per million reads. In this study, “Type” refers to the category representing the class of antibiotics that the resistance genes were capable of resisting. Each type encompassed one or more specific resistance genes, which were classified as “Subtypes”. For the annotation of MGEs, high-quality reads obtained from metagenomic sequencing were directly aligned against the MGE database [29] using the ARGs-OAP pipeline version 2.0. A read was annotated as plasmid-like, integron-like, transposase-like, insertion sequence transposase (*ist*)-like, or insertion sequence (IS)-like based on alignments performed with BLASTX, using an e-value threshold of 1 × 10^−7^ and a minimum amino acid similarity of 80%. Subsequently, the abundances of these MGEs were normalized to unit “ppm”, defined as one read per million reads.

### 2.6. Host Identification Analysis

These high-quality metagenomic reads were assembled into contigs using MEGAHIT [25]. Contigs with length ≥ 500 bp were selected as final assembling results. The open reading frame (ORF) was identified with METAProdigal (https://github.com/hyattpd/Prodigal, accessed on 1 October 2024), and the non-redundant gene catalog was then constructed using CD-HIT (v4.6.1) [30] with 90% sequence identity and 90% coverage. The abundance of each non-redundant gene was calculated by Salmon software [31]. The non-redundant gene sets were matched with the SARG database for ARG annotation using BLASTX, and then the annotated ARG contigs were taxonomic classified using the NCBI-NR database.

### 2.7. Identification of High-Risk ARGs

The risk of ARGs was assessed using an omics-based framework that included three criteria: (i) anthropogenic enrichment, (ii) mobility, and (iii) host pathogenicity [32]. Human-associated and mobile ARGs were classified as high-risk ARGs, which were further categorized into Rank I ARGs (meeting all three criteria, considered current threats) and Rank II ARGs (fulfilling criteria i and ii, regarded as future threats). Thus, Rank I and Rank II ARGs were identified from our data to evaluate potential health risks.

### 2.8. Statistical Analysis

Statistical comparisons between groups were carried out using nonparametric Kruskal–Wallis tests. Multiple testing corrections were applied using the Bonferroni method. Principal component analysis (based on the Bray–Curtis distance) and permutational multivariate analysis (Adonis) were conducted using the “vegan” package to illustrate the differences in ARGs and MGEs among groups. Additionally, Procrustes analyses with statistical significance tests were carried out using the same package. Network analysis was performed using Spearman correlation in combination with BH calibration. Results with a correlation coefficient greater than 0.8 and a *p*-value less than 0.05 were retained, and network diagrams were generated using the “ggraph” package.

### 2.9. Data Availability Statement

The raw sequence data generated in this study have been submitted to the Genome Sequence Archive (GSA) at the National Genomics Data Center, Chinese National Center for Bioinformation/Beijing Institute of Genomics, Chinese Academy of Sciences. The datasets were assigned accession numbers GSA: CRA018740, CRA018741, CRA018833, CRA018712, and CRA018950 and can be accessed via the following URL: https://ngdc.cncb.ac.cn/gsa (accessed on 1 October 2024).

## 3. Results

### 3.1. The Profiles of ARGs Involving Five Groups of Crows

**Figure 2:**
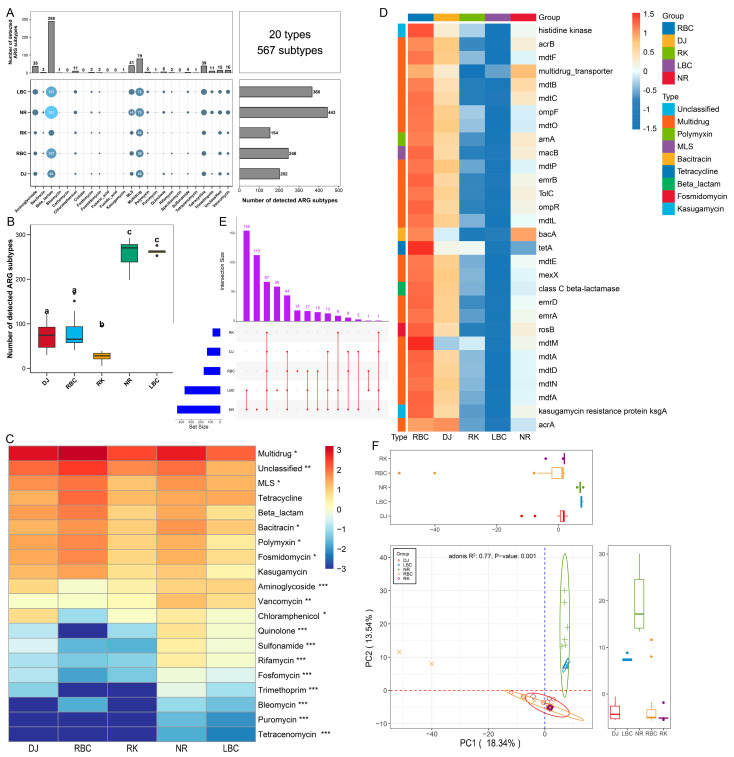
ARGs detected in five crow groups. (**A**) Combined statistical map of ARG types and subtypes for each group. (**B**) Comparison of the number of ARG subtypes across five groups. Statistically significant differences among groups are indicated by different lowercase letters. (**C**) Heatmap of ARG type abundances per group. Statistically significant differences are indicated as follows: ns, *p* > 0.05; * *p* < 0.05; ** *p* < 0.01; *** *p* < 0.001. (**D**) Heatmap of ARG subtype abundances per group. (**E**) Upset plot illustrating shared ARG subtypes across groups. (**F**) Principal component analysis plots based on Bray–Curtis distances for ARG subtypes.

A total of 20 types and 567 subtypes of ARGs were detected in the five crow groups, as illustrated in Figure 2A and Supplementary Table S1. Among these, the top 5 ARG types with the greatest number of subtypes were identified as follows: beta-lactam (comprising 288 subtypes), multidrug (79 subtypes), macrolide–lincosamide–streptogramins (MLS) (41 subtypes), tetracycline (39 subtypes), and aminoglycoside (38 subtypes). The distribution of ARG subtypes exhibited variation across the groups, with counts ranging from 154 to 443. Notably, the NR and LBC groups harbored the highest diversity of ARG subtypes, whereas the RK group possessed the lowest number of ARG subtypes, as shown in Figure 2B. The average abundances of ARG types varied between 0.005 and 642.327 ppm across the five groups, as depicted in Figure 2C. Among these, multidrug was identified as the most abundant type (642.327 ppm), followed by MLS (40.611 ppm), tetracycline (40.404 ppm), beta-lactam (29.783 ppm), and bacitracin (23.508 ppm). Tetracenomycin exhibited the lowest abundance (0.005 ppm). A Kruskal–Wallis test revealed that 17 ARG types showed significant differences in relative abundance among the five groups (Figure 2C). At the subtype level, the multidrug resistance gene *acrB* was found to be the predominant ARG subtype (64.79 ppm). Other notable subtypes included *bacA* (23.36 ppm) associated with bacitracin resistance, *macB* (21.46 ppm) linked to MLS resistance, *class C beta-lactamase* (16.73 ppm) related to beta-lactam resistance, and *tetA* (15.85 ppm) associated with tetracycline resistance, as illustrated in Figure 2D. Additionally, a total of 67 core ARG subtypes were found to be shared among the five groups, as shown in Figure 2E (Supplementary Table S2). The NR and LBC groups shared 155 ARG subtypes, with the NR group possessing 113 unique subtypes and the LBC group having 59 unique subtypes. Principal components analysis was conducted to cluster samples based on the relative abundance of ARG subtypes. Significant differences were observed among the five groups (Adonis, *R*^2^ = 0.77, *p* = 0.001; Figure 2F). In the analysis, the NR and LBC groups formed a single cluster, whereas the other three groups clustered separately.

### 3.2. The Profiles of MGEs Involving Five Groups of Crows

**Figure 3:**
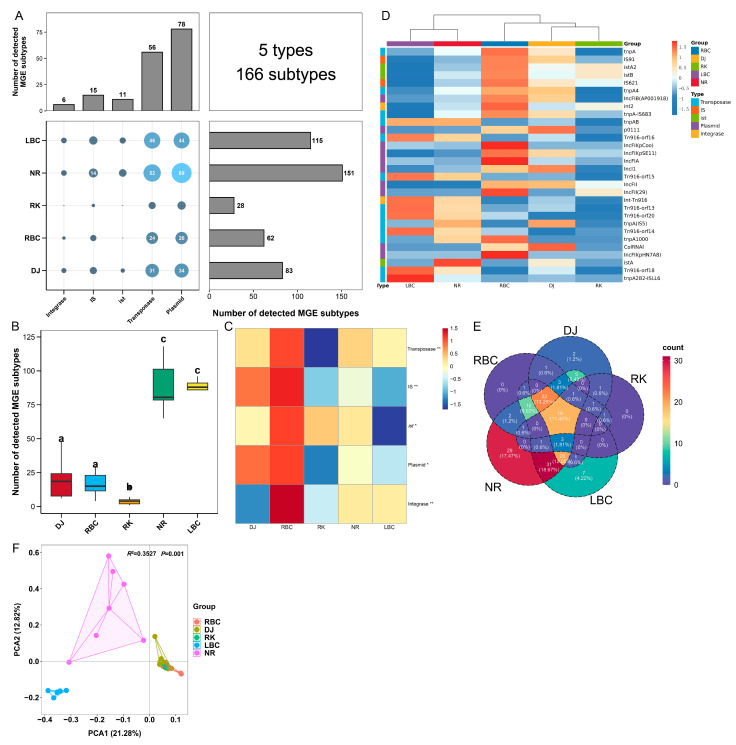
MGEs detected in five crow groups. (**A**) Combined statistical map of MGE types and subtypes for each group. (**B**) Comparison of the number of MGE subtypes across five groups. Statistically significant differences among groups are indicated by different lowercase letters. (**C**) Heatmap of MGE type abundances per group. Statistically significant differences are indicated as follows: ns, *p* > 0.05; * *p* < 0.05; ** *p* < 0.01. (**D**) Heatmap of MGE subtype abundances per group. (**E**) Venn plot illustrating shared MGE subtypes across groups. (**F**) Principal component analysis plots based on Bray–Curtis distances for MGE subtypes.

To investigate the potential mechanisms of ARG dissemination associated with different crow species, the diversity and abundance of MGEs were analyzed. A total of 5 MGE types, comprising 166 subtypes, were identified across the five groups, as shown in Figure 3A and Supplementary Table S3. Among these, plasmids and transposases were the predominant MGE types, with 78 and 56 subtypes detected, respectively. The distribution of MGE subtypes varied among the groups, with counts ranging from 28 to 151. Notably, the NR and LBC groups exhibited the highest richness of MGE subtypes, whereas the RK group displayed the lowest number of MGE subtypes, as illustrated in Figure 3B. The average abundances of MGE types varied between 14.39 and 367.56 ppm across the five groups, as depicted in Figure 3C. Transposase was identified as the most abundant MGE type, followed by IS, *ist*, Plasmid, and Integrase. Significant differences in relative abundance were observed for all five MGE types among the groups, as determined by the Kruskal–Wallis test. At the subtype level, *tnpA* (333.36 ppm) was found to be the predominant transposase subtype, while *IS91* (105.90 ppm), *istA2* (22.60 ppm), IncFIB(AP001918) (13.38 ppm), and *int2* (13.11 ppm) represented the most abundant subtypes for IS, *ist*, plasmid, and integrase categories, respectively, as shown in Figure 3D. Of the 166 MGE subtypes identified, the Kruskal–Wallis test indicated that 84 subtypes exhibited significant differences in abundance across the five groups. Additionally, a total of 19 core MGE subtypes were detected in all five groups. The NR and LBC groups possessed the highest number of unique MGE subtypes, with 29 unique subtypes in the NR group and 7 in the LBC group, as shown in Figure 3E. Furthermore, PCA revealed significant variation in MGE subtype composition among the five groups (Adonis, *R*^2^ = 0.35, *p* = 0.001; Figure 3F). In the analysis, the NR and LBC groups formed distinct clusters, while the other three groups grouped together into a single cluster.

### 3.3. The Profiles of Microbial Communities Involving Five Groups of Crows

**Figure 4:**
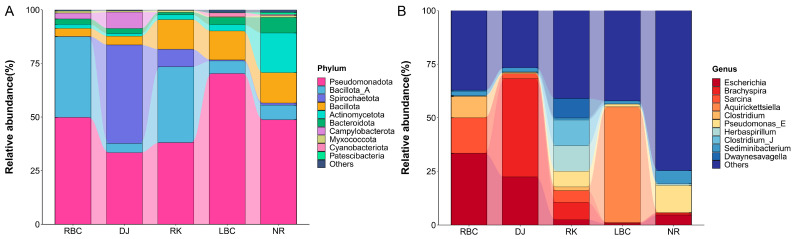
Composition of microbial communities in five crow groups. Taxonomic analyses conducted at the levels of (**A**) phylum and (**B**) genus.

The taxonomic compositions were analyzed at both the phylum and genus levels. A total of 132 bacterial phyla were identified across the five groups. At the phylum level, the microbial community was predominantly composed of Pseudomonadota (54.83%), followed by Bacillota_A (16.64%), and Bacillota (8.15%), as shown in Figure 4A. At the genus level, a total of 11,622 bacterial genera were identified across the five groups, and the top 10 most abundant genera were displayed in Figure 4B. The most abundant genera consisted of *Escherichia* (18.13%), *Brachyspira* (12.24%), and *Sarcina* (7.59%).

### 3.4. Correlations and Co-Occurrence Patterns Between Microbial Communities, ARGs, and MGEs

**Figure 5:**
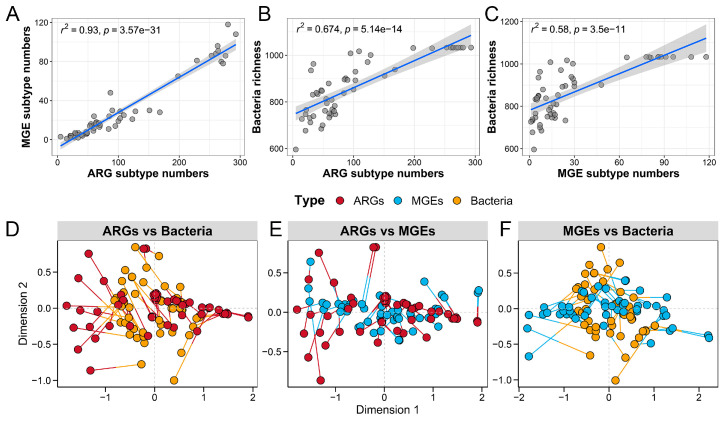
Interrelationships among gut microbes, ARGs, and MGEs. The correlations are shown between (**A**) ARGs and MGEs, (**B**) ARGs and gut microbes, and (**C**) gut microbes and MGEs. Procrustes analyses demonstrate the relationships between (**D**) ARGs and gut microbes, (**E**) ARGs and MGEs, and (**F**) gut microbes and MGEs.

To further explore how microbial composition influences resistome characteristics, we investigated the relationships between the gut microbial community and the antibiotic resistance profile. The analysis revealed significant positive correlations between the richness index of the gut microbiota and various components of the resistome. Specifically, strong correlations were observed between the gut microbiota richness and ARGs (*R*^2^ = 0.93, *p* < 0.001) (Figure 5A), ARGs and microbial diversity (*R*^2^ = 0.674, *p* < 0.001) (Figure 5B), as well as microbial diversity and MGEs (*R*^2^ = 0.58, *p* < 0.001) (Figure 5C). Additionally, Procrustes association analysis demonstrated significant associations among ARGs and microbes (M^2^ = 0.591, *p* < 0.001) (Figure 5D), ARGs and MGEs (M^2^ = 0.677, *p* < 0.001) (Figure 5E), and microbes and MGEs (M^2^ = 0.444, *p* < 0.001) (Figure 5F). These findings suggest that ARGs, MGEs, and gut microbes were closely interconnected, indicating that the composition of the microbial community played a key role in shaping the distribution of ARGs and MGEs within the fecal microbiome of the five crow groups.

**Figure 6:**
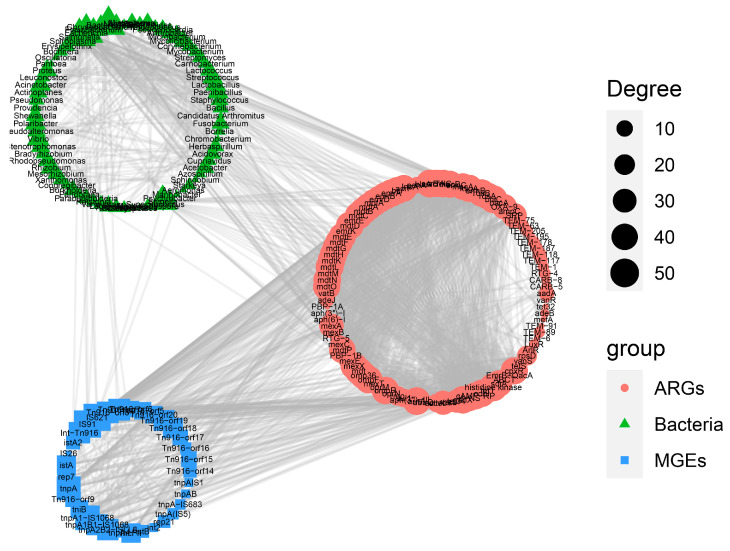
Network analysis showing the co-occurrence relationships among ARGs, MGEs, and gut microbes (at the genus level).

The co-occurrence patterns among ARGs, MGEs, and gut microbes were analyzed to illustrate their correlations within the crow microbiome (Figure 6). A total of 106 ARGs and 31 MGEs were found to have strong co-occurrence relationships with 74 gut microbial genera. Among these interactions, *IS91*, *IS621*, and *tnpA* were identified as the top three MGEs with the highest degree of connectivity, linking a large number of ARGs to specific bacterial genera. Within the 74 bacterial genera, *Escherichia*, *Bacteroides*, and *Salmonella* displayed the highest levels of connectivity, indicating their potential role as key hosts for ARGs and MGEs.

### 3.5. Identification of Bacterial Hosts of ARGs and MGEs

**Figure 7:**
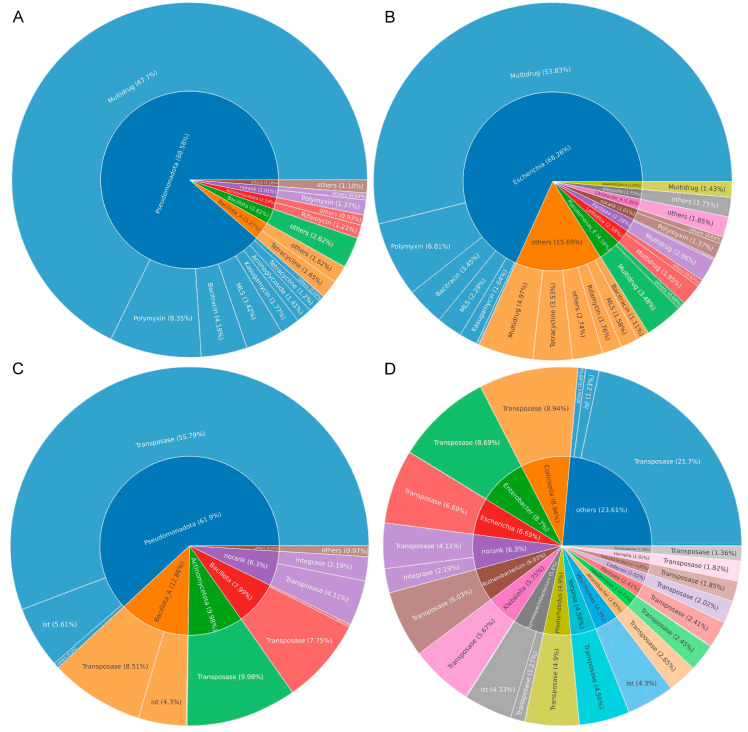
Hosts of the resistome. ARG hosts at the phylum (**A**) and genus (**B**) levels. MGE hosts at the phylum (**C**) and genus (**D**) levels. The inner circle indicates the annotation of phylum and genus hosts, while the outer circle represents the composition of ARG and MGE types.

After filtering out contigs with lengths less than 500 bp, a total of 3,357,068 contigs were assembled from all samples, with an N50 value of 1482 bp and the longest assembled contig reaching 1,055,132 bp. As shown in Figure 7A, the predominant phyla carrying ARGs in the crows were *Pseudomonadota* (88.58%), *Bacillota_A* (3.27%), and *Bacillota* (2.82%). At the genus level (Figure 7B), *Escherichia* (68.26%), *Pseudomonas_E* (4.16%), and *Lelliottia* (2.58%) were identified as the primary carriers of ARGs. For the gut microbiota harboring MGEs, the three most predominant phyla in crows were *Pseudomonadota* (61.9%), *Bacillota_A* (12.86%), and *Actinomycetota* (9.98%) (Figure 7C). At the genus level, *Collinsella* (8.94%), *Enterobacter* (8.7%), and *Escherichia* (6.69%) were identified as the top contributors of MGEs (Figure 7D). These findings suggested that both ARGs and MGEs could originate from multiple hosts, while a single host could simultaneously carry multiple ARGs and MGEs. Furthermore, the results highlighted that common opportunistic pathogens, such as *Escherichia*, *Pseudomonas_E*, and *Klebsiella*, served as the primary carriers of ARGs and MGEs in crows.

### 3.6. Resistance Mechanisms of ARGs in Crows

**Figure 8:**
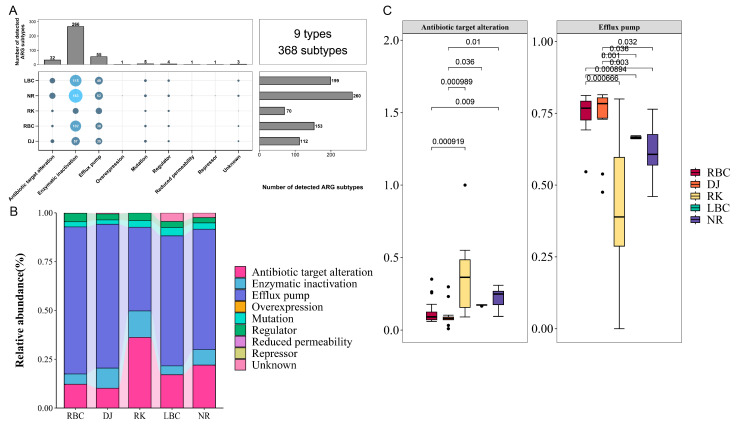
Resistance mechanisms in five crow groups. (**A**) Diversity of resistance mechanisms in each group. (**B**) Relative abundance composition of resistance mechanisms across groups. (**C**) Comparison of the top two resistance mechanisms abundances among groups.

A total of 9 types of antibiotic resistance mechanisms were detected, encompassing 368 subtypes of resistance genes (Figure 8A). The majority of ARGs in crows were associated with enzymatic inactivation (266 ARGs), efflux pump (55 ARGs), and antibiotic target alteration (32 ARGs) mechanisms. In terms of average abundance, efflux pumps accounted for approximately 73.76% of ARGs, followed by antibiotic target alteration (12.16%) and enzymatic inactivation (5.57%) (Figure 8B). These findings highlight the predominant roles of these mechanisms in shaping the antibiotic resistance profile of crows. We further examined the inter-group differences in antibiotic target alteration and efflux pump mechanisms across the five groups of crows. It was found that the RK group had a higher abundance of antibiotic target alteration, but a lower abundance of efflux pump mechanisms compared to the other groups (Figure 8C).

### 3.7. Detection of High-Risk ARGs in Crows

**Figure 9:**
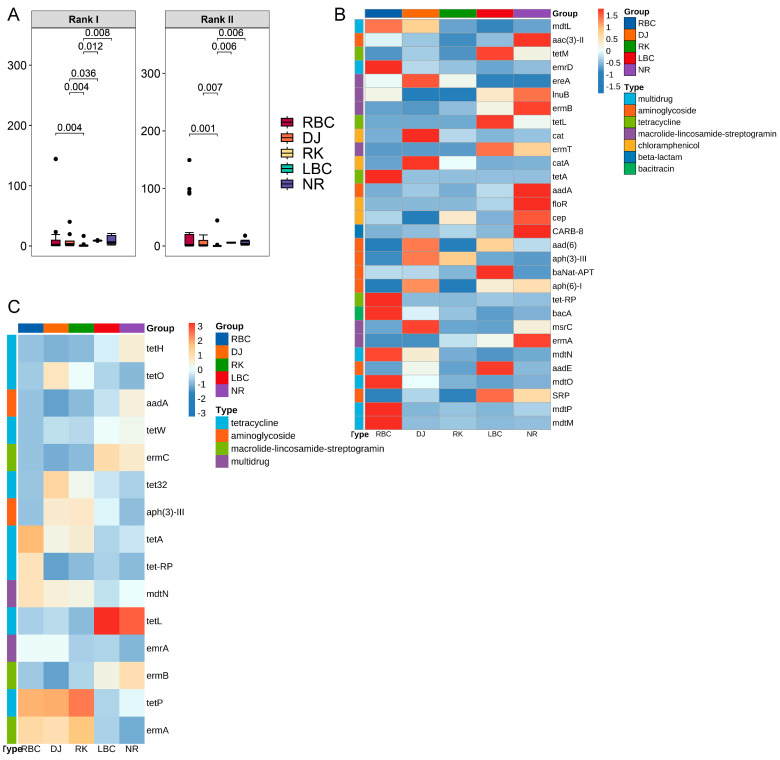
High-risk ARGs in five crow groups. (**A**) Comparison of inter-group differences in the relative abundance of high-risk ARGs at the rank I and rank II level. (**B**) Heatmap showing the abundances of rank I high-risk ARGs across groups. (**C**) Heatmap showing the abundances of rank II high-risk ARGs across groups.

A total of 81 ARG subtypes were classified as current threats (Rank I) (Supplementary Table S4), while 47 subtypes were categorized as future threats (Rank II) (Supplementary Table S5). Further differential analysis showed that among the five groups of crows, the RK group exhibited the lowest levels of both Rank I and Rank II risk ARGs (Figure 9A). Among the 81 Rank I ARG subtypes, the most prevalent types were tetracycline, multidrug, and MLS. Within the tetracycline category, *tetA*, *tet-RP*, and *tetM* were identified as the primary high-risk resistance genes of Rank I. Likewise, *mdtN*, *mdtO*, and *mdtM* were recognized as the key Rank I high-risk resistance genes in the multidrug category. Additionally, *ermB*, *msrC*, and *lnuB* were identified as the primary Rank I high-risk resistance genes within the MLS category. Among the top 30 Rank I high-risk ARGs based on relative abundance, 19 ARG subtypes were found to exhibit significant differences among the five groups (*p* < 0.05) (Figure 9B). For the 47 Rank II ARGs, the most prevalent types included tetracycline, MLS, and multidrug (Figure 9C). A total of 25 ARG subtypes within this category were also found to differ significantly among the five groups (*p* < 0.05).

## 4. Discussion

The habitats of wild birds were significantly intersected with those of humans and livestock, a factor that heightened the likelihood of horizontal transmission of ARB and ARGs across these groups [33]. To the best of our knowledge, this was the first study to employ metagenomic sequencing for a comparative analysis of ARGs among five prevalent crow species on the Qinghai–Tibetan Plateau.

In this study, five common omnivorous Corvidae species on the plateau, which did not undertake long-distance migrations, were selected. These species were observed as opportunistic foragers that had utilized predominantly anthropogenic food sources from refuse dumps, urban, suburban areas, and agricultural lands. Oravcová et al. employed strain isolation and cultivation techniques to analyze 75 VREfm (vancomycin-resistant *Enterococcus faecium*) isolates, finding that wild Corvidae birds had the potential to act as drivers for the global spread of vancomycin resistance [17]. Their investigation encompassed two species of Corvidae from our study: northern ravens (*Corvus corax*) and rooks (*Corvus frugilegus*). In recent years, traditional culturing techniques, quantitative PCR (qPCR), and metagenomics were utilized either individually or in combination to enhance the comprehension of antibiotic resistance [34]. Metagenomic sequencing facilitated the unbiased identification of a wide array of DNA sequences from samples under investigation. This approach did not necessitate specific target knowledge, thus enabling the discovery of ARG variants and novel ARGs that might have eluded detection by qPCR. The rapid advancement of metagenomic methodologies, which mapped the presence of ARGs within the comprehensive genomic landscape of samples, grew increasingly common in antibiotic resistance research [35]. Overall, the resistome analysis of five crow species identified 567 distinct ARG subtypes conferring resistance to 20 different antimicrobial substances. This comprehensive characterization provides critical insights into the diversity and distribution of ARGs within these avian populations, highlighting the complexity of their antibiotic resistome. Metagenomic data in this study had revealed that the resistomes were predominantly characterized by ARGs conferring resistance to multidrug, MLS, and tetracyclines, with notable relative abundances of ARGs associated with beta-lactam, and bacitracin as well. These five common types of ARGs were similarly detected in the gut resistome of another scavenger bird widely distributed on the Qinghai–Tibetan Plateau, the Himalayan vulture (*Gyps himalayensis*) [36]. This finding suggested a shared pattern of antibiotic resistance across different avian species in this ecological region. In contrast to domesticated species, crows were not directly exposed to antibiotic administration. Rather, their habitats acted as the main source of ARGs in their gut microbiomes [37]. Consequently, the intestinal resistome of crows provided insights into the levels of antibiotic and antibiotic resistance gene contamination present in their foraging habitats. When birds consume contaminated water or organisms, bacteria harboring ARGs may be introduced into their gut microbiomes. Despite the relatively low level of human disturbance on the Qinghai–Tibetan Plateau, recent studies had increasingly identified the presence of ARGs in water environments [38], soil [39], and wildlife [40] in this region, indicating the far-reaching impacts of human activities. Notably, even wild birds in remote and untouched regions, such as the polar areas, have been found to acquire antibiotic resistance [41]. In crows, multidrug resistance genes were the most abundant ARG type. Similarly, Li et al., found that multidrug resistance genes were also the most prevalent ARG type in wetlands on the Qinghai–Tibetan Plateau [42]. We hypothesized that the high abundance of multidrug resistance genes observed in the five crow species became the preferred choice for bacterial evolution due to their functional flexibility [43]. This adaptation was presumed to alleviate the energy burdens imposed on bacterial fitness in the absence of antibiotics, ultimately leading to the loss of unnecessary ARGs. In previous studies, efflux pumps have been identified as key contributors to multidrug resistance by actively expelling antibiotics from bacterial cells. These protein channels, embedded within the bacterial cell membrane, reduce the intracellular concentration of antibiotics, thereby diminishing their efficacy [44]. Specifically, in *Escherichia coli*, intrinsic resistance to multiple antimicrobial agents was produced through the expression of the three-component multidrug efflux system AcrAB-TolC [45]. In this system, AcrB acted as a proton-motive-force-dependent transporter located in the inner membrane, whereas AcrA and TolC functioned as accessory proteins positioned in the periplasm and outer membrane, respectively. Further analysis revealed that *AcrB* was the most abundant subtype within the multidrug resistance category in our study, highlighting its significant role in this context. Our study also identified that the MLS resistance gene *macB*, the beta-lactam resistance gene *class C beta-lactamase*, and the tetracycline resistance gene *tetA* exhibited high abundance across various samples. The acquisition of these subtypes drew significant attention due to the ensuing challenges they posed for resistance management. Notably, the *class C beta-lactamase* gene conferred resistance to a broad spectrum of beta-lactam antibiotics, such as penicillins, cephalosporins, and carbapenems, which in turn complicated the treatment and control of common bacterial infections [46]. Furthermore, the extensive use of tetracycline was also likely to result in the enrichment of tetracycline resistance genes (*tet*) within the environment. Extensive large-scale research is required to explore the contribution of habitat environments to the gut resistome of crows in the future. This study also revealed interspecies differences in the composition of resistance genes among the five crow species examined. We speculated that these differences might be related to variations in the dietary habits and habitat environments of the crow species. However, this study lacked dietary habits and background levels of antibiotic contamination in these habitats for the species, which prevented us from exploring the specific reasons for these differences. This limitation represents one of the shortcomings of our study.

The primary resistance mechanisms identified in the crow resistome included antibiotic efflux pumps, alteration of antibiotic targets, and enzymatic inactivation. These mechanisms were similarly detected as the main strategies in the Himalayan vultures [36]. The use of efflux pumps for antibiotic extrusion was identified as an effective resistance strategy against varied environmental pressures. Genes providing antibiotic resistance through these mechanisms were well-suited to different ecosystems and exhibited a tendency for horizontal transfer between bacterial hosts. Blanco et al. reported that multidrug resistance frequently relied on efflux pump mechanisms [47]. In our analysis, efflux pumps were identified as the dominant resistance mechanism in crows, a finding we speculated was due to the higher frequency of multidrug resistance genes within the crow resistome. Additionally, the second ARG resistance mechanism identified in crows involved alterations in antibiotic targets. These changes hindered the binding of antibiotics to bacterial cells, preventing them from inhibiting crucial cellular components, while preserving the normal metabolic function of the target. Antibiotics typically function by binding tightly to specific bacterial targets, enabling them to penetrate bacteria and exert their antimicrobial effects. However, modifications to these targets can impair the binding efficiency of antibiotics, thereby reducing their ability to inhibit bacterial growth and activity [48]. In Gram-positive bacteria, the inactivation of antibiotics is an irreversible resistance mechanism that primarily occurs through two processes: enzymatic degradation of the drug or the addition of a chemical group to the antibiotic [49]. This mechanism has often been observed in resistance to beta-lactam, aminoglycoside, and tetracycline antibiotics. Notably, within the crow resistome, the abundance of resistance genes for these two classes of antibiotics was relatively high. A diverse array of bacteria utilizes a spectrum of mechanisms to endure the selective pressures imposed by antibiotics. Comprehending these mechanisms is fundamental for the innovation of new strategies to counteract antibiotic resistance. This includes not only the development of novel antibiotics but also the exploration of combination therapies, as well as other cutting-edge treatments.

ARGs can be transferred among species in the wild through various MGEs. This mechanism facilitates the spread of antibiotic resistance across different organisms and ecosystems, highlighting the complexity of managing and mitigating resistance development. In our study, we identified a total of 5 types of MGEs, comprising 166 subtypes, across five distinct groups. The sequences of MGEs responsible for horizontal gene transfer, including *tnpA* (transposase), *IS91* (IS), *istA2* (*ist*), IncFIB(AP001918) (plasmid), and *int2* (integrase), were present in high abundance. *TnpA*, particularly within the ubiquitous and abundant Tn3-family of transposase genes, highlights the evolutionary significance of transposable elements in generating genetic diversity. By means of its replicative transposition mechanism, *tnpA* promotes both the emergence of new pathogens and the spread of antimicrobial resistance, including against last-resort antibiotics such as carbapenems and colistin [50]. *IS91* functioned as the prototype element of a bacterial insertion sequence family that transposed through a rolling-circle mechanism. Analysis revealed that *IS91* family elements were frequently located adjacent to genes associated with pathogenicity and virulence [51]. This positioning suggested a significant role for these elements in the dissemination and evolution of virulence and pathogenicity traits. Notably, we observed a high abundance of the IncFIB (AP001918) plasmid in the crow resistome. Genomic analysis revealed that this plasmid carried the virulence genes *ehxA* and *etpD*, known contributors to strain pathogenicity [52]. These findings suggested that IncFIB (AP001918) likely contributed to the strain’s virulence and potential for horizontal gene transfer (HGT). Johnson et al. previously reported that *IncFIB* was the most prevalent type in avian, human, and poultry meat isolates in the US [53]. However, there were variations in plasmid replicons and colicin-related genes across different *Escherichia coli* sources. Our results highlighted the significance of IncFIB (AP001918) within the crow population, indicating its potential role in facilitating the spread of virulence factors and antimicrobial resistance across species boundaries. Meanwhile, co-occurrence analysis also revealed many high-degree-connected ARGs, MGEs, and gut microbial genera. These were also identified as critical targets that warranted greater attention in future research. ARGs linked to MGEs can readily transfer between hosts within a microbial community, posing significant risks to human health. In this regard, there is an urgent need for ongoing surveillance of these genes in crow populations. Further studies are necessary to determine if bacteria can obtain these ARGs via horizontal gene transfer and consequently evolve antibiotic resistance.

Moreover, an analysis was conducted on the hosts of ARGs and MGEs within crow populations. The host range included several genera of opportunistic pathogens. For example, *Escherichia* and *Collinsella* were the primary hosts of ARGs and MGEs, respectively. Previous studies have indicated that *Escherichia coli* is among the foremost carriers of ARGs within the gut microbiota [54]. *Escherichia coli* harboring virulence factor genes and numerous ARGs may present a significant threat to human health. For instance, an *E. coli* outbreak occurred in Germany in 2011, which led to acute diarrhea, abdominal pain, and even fatalities among the affected population [55]. The genus *Collinsella*, belonging to the family Coriobacteriaceae, was classified as pathobionts. Studies have demonstrated that exposure to high levels of various metals led to an increased abundance of *Collinsella*, a pro-inflammatory bacterium [56]. This bacterium was found to reduce the expression of tight-junction proteins in enterocytes and induce a leaky gut condition [57]. These features were associated with metabolic endotoxemia, indicating a significant role of *Collinsella* in the pathogenesis of intestinal barrier dysfunction and related metabolic disorders. Therefore, future research should employ culture-based methods to isolate and characterize gut-associated antibiotic-resistant pathogenic strains from crows. This work will facilitate a deeper understanding of the evolution of ARGs and aid in the identification of effective antibiotics capable of eliminating these pathogenic bacteria.

Previous studies demonstrated that the host resistome is influenced by its bacterial communities [58]. An important connection between the resistome and the microbial composition in crows was established. This observed linkage implies that variations in the microbial community structure within crow populations might affect their harboring of ARGs. We identified a total of 132 bacterial phyla and 11,622 bacterial genera in crows, among which *Escherichia*, *Brachyspira*, and *Sarcina* were predominantly represented. The gut microbiota of crows was found to encompass numerous pathogenic or opportunistic pathogenic microorganisms. We hypothesized that this prevalence of pathogens could be attributed to the crows’ diverse dietary habits and high adaptability, allowing them to thrive across a wide range of habitats. We hypothesized that if opportunistic pathogens within the crow gut microbiota acquire ARGs through horizontal gene transfer, this could pose a potential risk for zoonotic infections that might be difficult to treat in affected species, including humans and other animals. The risk assessment revealed that within the crow resistome, there were 81 ARGs classified at Rank I and 47 ARGs at Rank II. These “high-risk” ARGs are linked to “current threats” characterized by their significant potential to introduce multidrug-resistant pathogens, and “future threats” which may transfer to pathogens as novel resistance mechanisms develop. Additional studies are needed to investigate the antibiotic susceptibility profiles of these opportunistic pathogen species and to examine the horizontal gene transfer of “high-risk” ARGs among different strains.

Overall, our study enhances the understanding of resistome characteristics across five species of Corvidae and their variations among different environments. Future research should aim to determine the background levels of antibiotic contamination in the water and soil of their habitats, as well as to identify the transmission pathways of ARGs associated with these bird species.

## 5. Conclusions

These findings significantly enhanced the assessment of public health risks associated with antibiotic resistance harbored by wild Corvidae birds, an area that had previously received limited attention. The data provided critical insights into the prevalence and distribution of resistance genes within these avian populations, contributing to a more comprehensive understanding of their potential role in the dissemination of antibiotic resistance. By elucidating the dynamics of resistance carriage in wild Corvidae birds, this research underscored the need for targeted surveillance and intervention strategies to mitigate the broader public health implications of antibiotic resistance transmission across species and ecosystems. Furthermore, integrating transcriptome data with culture-based experiments will be essential to enhance metagenomic approaches, thereby offering a deeper understanding of the risks associated with ARGs and their interactions with host microorganisms.

## Data Availability

The raw sequence data generated in this study have been submitted to the Genome Sequence Archive (GSA) at the National Genomics Data Center, Chinese National Center for Bioinformation/Beijing Institute of Genomics, Chinese Academy of Sciences. The datasets were assigned GSA accession numbers CRA018740, CRA018741, CRA018833, CRA018712, and CRA018950 and can be accessed via the following URL: https://ngdc.cncb.ac.cn/gsa (accessed on 1 September 2024).

## Supplementary Materials

The following supporting information can be downloaded at https://www.mdpi.com/article/10.3390/vetsci12040297/s1. Table S1. The types and subtypes of ARGs for each sample, with the numerical values expressed as one read per million reads (ppm); Table S2. List of 67 core ARGs subtypes; Table S3. The types and subtypes of MGEs for each sample, with the numerical values expressed as one read per million reads (ppm); Table S4. The types and subtypes of high-risk ARGs (Rank I) for each sample, with the numerical values expressed as one read per million reads (ppm); Table S5. The types and subtypes of high-risk ARGs (Rank II) for each sample, with the numerical values expressed as one read per million reads (ppm).
